# The Treatment of Intestinal Cancer

**Published:** 1893-06-17

**Authors:** 


### ROYAL FREDERICK HOSPITAL, COPENHAGEN.

### The Treatment of Intestinal Cancer.

Intestinal cancer, even if we leave out of consideration that which affects the rectum, is so frequent and so terrible from the suffering which it causes, that the gravest operations have been regarded as justifiable. Colostomy, or even resection of the intestine, with reduction at the same time, have yielded very unsatisfactory results. Dr. Oscar Bloch has, however, devised and carried out a new method which avoids many of the risks and gives the patient a much better prospect. He endeavours to diagnose and treat the disease as early as possible. The operation is divided into three different stages done at different times. (1) The abdomen is opened under the usual precautions by an incision some eight centimetres in length. If, as frequently happens, the growth is in the sigmoid flexure, the incision is made in the left iliac fossa and the intestine drawn out which contains the cancerous nodule, as well as healthy parts above and below it. Adhesions, if necessary, are broken down, and the loop of gut is fixed as in Maydl's plan for colostomy by means of a glass rod passed through the mesentery, so that the entire portion remains in sight lying on the wall of the abdomen. The wound is reduced in size by catgut sutures and covered with iodoform gauze.

A transverse incision is made in the intestine by the thermocautery to the extent of four or five centimetres, some centimetres above the narrowest part of the stricture, and a large drainage tube is inserted into the lower part of the intestine. Iodoform gauze is packed round the base of the whole of the extruded piece of gut, and a dressing of sterilized gauze and absorbent wool applied. The entire operation does not last more than thirty minutes.

(2) When firm adhesions between the exposed portion of gut and the abdominal opening are formed, Dr. Bloch goes on to resection of the cancerous part. The healthy ends are united by sutures. This is done, as he claims, without the slightest danger to the patient, for this part of the operation is performed outsi le the abdomen, and the peritoneal cavity is not opened. Opium is given to prevent the action of the bowels before union is complete. Afterwards the intestine may be watched for a time for any appearance of recurrence. This was done in one case where some sutures had given way, and an additional reason for delay was therefore present, for 114 days. (3) If the action of the bowels is then normal, and there is no evidence of recurrence, the artificial hernia is reduced and the wound closed.

In comparing this plan with other methods of operation, Dr. Bloch refers to the results obtained elsewhere (*a*) under radical treatment by (1) excision of the growth, by resection of the gut, and immediate reduction. Out of twenty-nine recorded cases, only twelve were successful, and in five of these a fistula remained, and not less than six showed signs of recurrence. The mortality is chiefly from peritonitis, either at the time or from rupture of sutures. Hence also the importance of forming a temporary artificial anus. Where (2) resection has been performed without an attempt at reuniting the ends of the intestine, the mortality seems equally great, and recurrence carries off part of the survivors. Even in (*b*) merely palliative treatment, that is, the formation of an artificial anus without any excision of the growth, the mortality is considerable, and as this must be inevitably undertaken in many cases, there is very little, if any, increase of danger in carrying out Dr. Bloch's method, which gives a prospect of permanent cure if done at an early stage.

By this method of resection outside the abdomen, the great danger from collapse, peritonitis, and failure of sutures, is very greatly diminished. Peritonitis is guarded against, provided the peritoneal cavity is closed before opening the intestine. A simple opening into the gut at the first stage of the operation, if necessary, causes clearly less danger of the entrance of pus and fæces into the peritoneum than a resection does. If ileus is not present, the gut need not be opened at all before it has formed adhesions to the peritoneal envelope of the abdominal wall, when the risk of peritonitis is almost nil. In the second stage, when all is quiet, and defæcation by the artificial anus has become normal, resection of the carcinoma from the neighbouring healthy parts of the gut can be done without haste, even without anæsthetics, and indeed, with little danger to the patient, the peritoneum being entirely closed. It is then also possible to watch the patient as long as necessary, to guard against the risk of necrosis of the ends of the resected intestine, and to see that there is no recurrence. Finally, the patient can decide for himself whether he will undergo the danger of closing the artificial anus. As this danger chiefly depends on the failure of sutures, we may perform even this outside the abdomen, suturing the artificial opening, and when the normal passage of the motions along the intestine is restored the latter may be replaced in the abdomen in safety. However, if the gut be much contracted it may not be possible to carry out this closing of the artificial anus completely in the method suggested.

If there is a long mesentery at the spot where the growth is found, as there usually is, there is no difficulty; but if the mesentery is infiltrated, and we nevertheless think that there is a reasonable prospect of good from radical treatment, we may incise the peritoneum at the point where it passes from the intestine to the abdominal wall, and detach the intestine by the aid of the finger. In 80 out of 145 recorded cases the growth was situated in the sigmoid flexure where the mesentery is specially long.

Finally, as to metastases, Dr. Bloch holds that there is good reason to think that at an early stage the local affection is the only one, and that an operation then may effect a permanent cure. It is, however, most difficult to say when this stage is passed. Hence the vast importance of an early diagnosis and treatment of the disease.

